# Risk Factors for Peritonitis Associated With Endoscopic Ultrasound‐guided Hepaticogastrostomy

**DOI:** 10.1002/deo2.70376

**Published:** 2026-08-14

**Authors:** Junya Sato, Hirotoshi Ishiwatari, Hiroki Sakamoto, Takuya Doi, Masahiro Yamamura, Kazunori Takada, Masao Yoshida, Sayo Ito, Noboru Kawata, Kenichiro Imai, Kinichi Hotta, Hiroyuki Ono

**Affiliations:** ^1^ Division of Endoscopy Shizuoka Cancer Center Shizuoka Japan; ^2^ Department of Gastroenterology St. Marianna University School of Medicine Kawasaki City Japan; ^3^ Division of Pancreatobiliary Medicine Shizuoka Cancer Center Shizuoka Japan

**Keywords:** biliary tract, drainage, endoscopic ultrasonography, endoscopy, peritonitis

## Abstract

**Background and Objectives:**

Endoscopic ultrasound‐guided hepaticogastrostomy (EUS‐HGS) is an alternative therapeutic option for unsuccessful endoscopic retrograde cholangiopancreatography in patients with malignant biliary obstruction. Procedure‐related adverse events (AEs), such as bile leakage and peritonitis, can lead to prolonged hospitalization and delay the initiation of chemotherapy for underlying malignancies. This study aimed to identify factors associated with the development of peritonitis following EUS‐HGS.

**Methods:**

Consecutive patients who underwent initial EUS‐HGS between July 2016 and November 2022 were retrospectively evaluated. We assessed the preprocedural characteristics (cholangitis and duodenal invasion) and intraprocedural technical factors (small bile duct diameter, short hepatic parenchymal distance at puncture, antegrade stenting, and the use of a plastic stent for HGS). Multivariate logistic regression analysis was conducted to evaluate the factors associated with peritonitis after EUS‐HGS.

**Results:**

A total of 188 patients were included. The cause of biliary obstruction was malignancy in 96% of the cases, with the majority being pancreatic cancer. The rate of early AEs was 15% and included peritonitis (7%), pancreatitis (3%), cholangitis (3%), cholecystitis (1%), and bleeding (1%). Multivariable logistic regression analysis revealed that the diameter of a punctured bile duct ≤3 mm (odds ratio, 4.0; 95% confidence interval, 1.1–15; *p* = 0.039) independently increased the risk of peritonitis after EUS‐HGS.

**Conclusions:**

A punctured bile duct diameter of ≤3 mm was identified as a significant independent risk factor for the development of peritonitis after EUS‐HGS.

**Trial Registration**: N/A

## 1 Introduction

Malignant biliary obstruction (MBO) leads to obstructive jaundice and cholangitis, impairing patients’ quality of life. Biliary drainage is the primary treatment for these complications, and the methods, routes, efficacy, and safety of the drainage are critical issues in the management of MBO. Endoscopic retrograde cholangiopancreatography (ERCP)‐guided drainage is the current standard treatment for MBO; however, ERCP can be difficult or infeasible in certain cases, such as those complicated by duodenal obstruction [1]. Endoscopic ultrasonography (EUS)‐guided biliary drainage was first described in 2001 [2]. Currently, it is primarily used as an alternative therapeutic option in cases of unsuccessful ERCP. Primary EUS‐guided biliary drainage routes include EUS‐guided hepaticogastrostomy (EUS‐HGS) and choledochoduodenostomy (EUS‐CDS). Although EUS‐HGS and EUS‐CDS involve different puncture routes, a randomized controlled trial reported the noninferiority of EUS‐HGS to EUS‐CDS in terms of technical success rates (87.5% vs. 82.6%) [3]. The same study also demonstrated no significant differences in the clinical success rates, treatment‐related adverse events (AEs), or stent patency between the two groups, suggesting that both EUS‐HGS and EUS‐CDS are effective biliary drainage methods. However, EUS‐HGS is often the only feasible option for patients with duodenal stenosis. Furthermore, some reports indicated that EUS‐HGS is safer and provides longer stent patency than EUS‐CDS in patients with duodenal invasion [4]. In recent years, the utility of EUS‐HGS has been suggested not only for distal biliary obstruction but also for hilar biliary obstruction and drainage in patients with a surgically altered anatomy [5, 6, 7, 8]. The advantages of EUS‐HGS include the ability to create an internal fistula in a single session and the virtual absence of treatment‐related pancreatitis, which is a common complication of ERCP. However, as the procedure creates a fistula between the gastrointestinal tract and bile duct, AEs that are rare with ERCP, such as bile leakage and peritonitis, can occur in 2.2%–21% of cases [9, 10, 11, 12, 13, 14]. Bile leakage and peritonitis can lead to prolonged hospitalization and delayed initiation of chemotherapy for underlying malignancies. If the condition is severe, invasive interventions, including surgery, may be necessary. To reduce these AEs, the current guidelines recommend the use of metal stents (MSs) over plastic stents (PSs) for EUS‐HGS [15]. Given their self‐expanding properties, MSs are theoretically expected to prevent bile leakage into the fistula tract. However, peritonitis after EUS‐HGS has been reported in 4.6–6.2% of cases, even with the use of an MS, highlighting the need to identify other preventive measures and clarify the risk factors for these AEs [16, 17]. This study aimed to identify the factors associated with the development of postprocedural peritonitis following initial EUS‐HGS.

## 2 Methods

### 2.1 Study Design

This retrospective study was conducted at the Shizuoka Cancer Center, a high‐volume tertiary referral facility that specializes in cancer treatment. We systematically reviewed and recruited consecutive patients from electronic medical records and a prospectively maintained EUS‐guided biliary drainage database. The patients underwent initial EUS‐HGS between July 2016 and November 2022. We excluded five patients from the final analysis: four in whom the EUS‐HGS procedure was technically unsuccessful, and one patient who underwent simultaneous EUS‐HGS performed on both intrahepatic bile ducts in segments 2 (B2) and 3 (B3). The primary objective of this study was to identify the risk factors for post‐EUS‐HGS peritonitis using multivariate analysis. Peritonitis was defined as the concurrent presence of all the following criteria within seven days of the procedure, necessitating either a prolonged hospital stay or further intervention: (1) elevated levels of inflammatory markers on blood tests or the presence of fever; (2) onset of new abdominal pain; and (3) newly observed fluid collection within the abdominal cavity on computed tomography (CT). Secondary endpoints included all other AEs that were defined based on the established lexicon of the American Society for Gastrointestinal Endoscopy. The severity grading of these AEs was determined in accordance with published guidelines [18]. Institutional ethical approval for this study was obtained from our facility's review board (J2022‐218), and the study adhered to the principles outlined in the Declaration of Helsinki. Before EUS‐HGS, all patients provided informed consent for the procedure (Supporting Information).

### 2.2 EUS‐HGS Procedure

The EUS‐HGS procedures were performed by or under the direct supervision of experienced endoscopists who had independently performed over 30 EUS‐HGS procedures before the commencement of this study. This technique involves inserting a curved linear‐array echoendoscope into the stomach. After ensuring that no intervening vessels were present on color Doppler ultrasonography, B2 or B3 was accessed using either a 19‐ or 22‐gauge needle. The bile duct for puncture was selected at the discretion of the operator based on which site allowed for the safest access. A 0.025‐ or 0.018‐inch guidewire was advanced into the bile duct. An ERCP catheter was introduced for bile aspiration because our previous data indicated that aspiration might mitigate the rate of AEs [19]. In some cases involving a surgically altered anatomy and the absence of duodenal obstruction, an antegrade stenting procedure was first performed across the bile duct obstruction site before the final HGS stent was placed. The uncovered MSs used for antegrade stenting were the Zilver 635 (Cook Medical, Bloomington, IN, USA) and BileRush (Piolax, Kanagawa, Japan). Tract dilation was performed immediately before the placement of the HGS stent. Dilation was occasionally omitted if a PS was used or if the MS delivery system had a diameter of less than 7.5 Fr. The choice of the dilation device was contingent on the stent type; a bougie dilator was used for PSs, whereas a balloon catheter or cystotome was the preferred instrument for MSs. Representative devices included a 7F bougie dilator (ES dilator; Zeon Medical, Tokyo, Japan), 4‐mm balloon catheter (REN Biliary Balloon Dilation Catheter; Kaneka, Osaka, Japan), 6F cystotome (Cysto‐Gastro‐Set; Endo‐Flex GmbH, Voerde, Germany), and Tornus ES (Asahi Intecc, Aichi, Japan). Finally, the MS or PS was advanced across the tract, establishing a bypass between the intrahepatic duct and stomach. The MSs primarily used for HGS were bare‐end covered stents, namely the Niti‐S biliary S‐type (Taewoong Medical, Gimpo, South Korea), EGIS biliary (S&G Biotech, Yongin‐si, Korea), and HANAROSTENT Biliary Partial Cover Benefit (Boston Scientific, Marlborough, MA, USA). Most MSs range from 6 to 10 mm in diameter and 10 to 12 cm in length. The sole PS utilized for HGS was the Through‐and‐Pass Type IT (Gadelius Medical, Tokyo, Japan), available in 7F diameter and 14 cm in length. Our institutional policy for stent selection was to use metallic stents for HGS in cases of malignant biliary obstruction, while PSs were used for benign diseases or cases with post‐surgical reconstructed anatomy. In cases where antegrade stenting was also performed, the choice between a metallic or PS for the HGS site was left to the operator's discretion.

### 2.3 Postprocedural Management

All EUS‐HGS procedures were performed during hospitalization. Blood tests were performed on the first day after the procedure. Oral intake was initiated on the second postprocedural day after confirming the absence of AEs. If patients developed fever or new abdominal pain, a CT scan was promptly performed to assess for AEs.

### 2.4 Statistical Analysis

Categorical variables were compared using Fisher's exact test. The Mann–Whitney U‐test was used to compare continuous variables. Multivariate logistic regression analysis was performed to evaluate the influence of various factors on the occurrence of peritonitis after EUS‐HGS. To date, no definitive factors associated with the development of peritonitis after EUS‐HGS have been identified. Therefore, we explicitly evaluated a comprehensive range of factors. Systemic and preprocedural patient factors included cholangitis (suggestive of infected bile), hyperbilirubinemia (predictive of severe cholestasis), and diabetes mellitus (which may induce an immunocompromised state). We also considered anatomical and pathological conditions that may elevate intragastric pressure by impairing intestinal peristalsis, including surgically altered anatomy, MBO, duodenal invasion, peritoneal dissemination, and ascites. Intraprocedural technical factors potentially contributing to bile leakage were examined, specifically small bile duct diameter, additional fistula dilation, use of an electrocautery device, and short hepatic parenchymal distance at the puncture site. Finally, we assessed factors related to management and device selection, including the omission of intraprocedural bile aspiration and antegrade stenting (which may reduce bile leakage by decompressing the bile duct), and the use of a PS for HGS (which may allow bile leakage through the gap at the fistula tract). For exploratory analysis, factors with a *p*‐value of less than 0.1 in the univariate analysis were subsequently integrated into the multivariate model. Statistical significance was set at *p* < 0.05 for all tests. All statistical computations were performed using EZR version 4.0.2 (Saitama Medical Center, Jichi Medical University, Saitama, Japan), which functions as a graphical user interface for the R software (R Foundation for Statistical Computing, Vienna, Austria).

## 3 Results

Between 2016 and 2022, 188 patients underwent initial EUS‐HGS. Patient characteristics are shown in Table 1. The cause of biliary obstruction was malignant in 96% of the cases, with the majority being pancreatic cancer. Surgically altered anatomy was observed in 32% of patients, preprocedural cholangitis in 63%, duodenal invasion in 63%, ascites in 18%, and peritoneal carcinomatosis in 23%. The procedure‐related factors are summarized in Table 2. The median diameter of the punctured bile duct was 6 mm, and the median distance of the hepatic parenchyma along the puncture tract was 28 mm. The median bile volume aspirated before tract dilatation was 25 mL. Additional fistula dilatation was performed in 168 patients. The devices used for fistula dilation were as follows: electrocautery dilator alone (*n* = 67), dilation balloon alone (n = 32), dilation catheter alone (*n* = 30), combination of balloon and electrocautery (*n* = 14), combination of catheter and electrocautery (*n* = 4), combination of catheter and balloon (*n* = 14), and a combination of all three (*n* = 3). Antegrade stenting was performed in 57 cases. For HGS, PSs were used in 60 cases, and MSs were used in 128 cases. Early AEs were observed in 15% of the patients (Table 3). The breakdown of AEs included peritonitis in 13 patients, pancreatitis in six, cholangitis in five, cholecystitis in two, and bleeding in two. Five cases of peritonitis resolved with antibiotic administration alone. Among the remaining patients, in addition to antibiotics, duodenal stent placement was performed in four patients who had concurrent duodenal stenosis to reduce intragastric pressure. Furthermore, additional placement of an MS for HGS was performed in one case because of bile leakage caused by damage to the covered membrane of the initial MS. In another preoperative case, in which a PS was initially placed for HGS, the stent became clogged owing to food impaction, prompting the addition of antegrade stenting using a MS. Surgery was required in two cases due to worsening peritonitis despite conservative treatment: one case involving biliary stricture due to postoperative recurrence of gastric cancer and one case involving biliary and duodenal strictures due to pancreatic cancer where antegrade stenting was difficult. In the case of pancreatic cancer, surgery was performed because of suspected peritonitis caused by bile leakage; however, intraoperative findings revealed extensive peritoneal seeding, and ascites analysis confirmed the diagnosis of carcinomatous peritonitis. Univariate analysis was performed to identify factors associated with the development of peritonitis, and factors with a *p*‐value <0.1 were included in the multivariate analysis. Consequently, a punctured bile duct diameter of ≤3 mm was identified as an independent risk factor associated with the development of peritonitis (Table 4).

**TABLE 1:** Baseline characteristics.

Characteristic	Result (*n* = 188)
Median age, years (range)	71 (26–95)
Male/female	106/82
Performance status (0–1/2–4)	117/71
Cause of biliary obstruction, *n*	
Malignant	181 (96%)
Pancreatic/biliary/other neoplasms	100/32/49
Benign	7 (4%)
Hepaticojejunostomy stricture	6
Bile duct stone	1
Site of biliary obstruction, *n*	
Distal/hilar/other	149/30/9
Surgically altered anatomy, *n*	60 (32%)
Preprocedural cholangitis, *n*	116 (63%)
Duodenal invasion, *n*	119 (63%)
Ascites, *n*	33 (18%)
Peritoneal dissemination, *n*	43 (23%)

**TABLE 2:** Procedural factors of endoscopic ultrasound‐guided hepaticogastrostomy (EUS‐HGS).

Factor	Result (*n* = 188)
Needle size, 19‐G/22‐G	159/29
Punctured bile duct, B2/B3	83/105
Diameter of punctured bile duct, mm, median (range)	6 (2‐13)
Distance to hepatic parenchyma, mm, median (range)	28 (2–53)
Quantity of aspirated bile, mL, median (range)	25 (0–200)
Tract dilation using dilation devices, *n*	168 (89%)
Use of electronic cautery catheter for dilation, *n*	87 (46%)
Antegrade stenting, *n*	57 (30%)
Stent placed HGS, plastic stent/metal stent	60/128
Procedure time, min, median (range)	32 (9‐121)

Abbreviations: EUS, endoscopic ultrasound; HGS, hepaticogastrostomy.

**TABLE 3:** Early adverse events.

Event	Result (*n* = 188)
All, *n*	28 (15%)
Peritonitis, *n*	13 (7%)
I/II/IIIa/IIIb/IVa/IVb/V	0/5/6/2/0/0/0
Pancreatitis, *n*	6 (3%)
I/II/IIIa/IIIb/IVa/IVb/V	0/6/0/0/0/0/0
Cholangitis, *n*	5 (3%)
I/II/IIIa/IIIb/IVa/IVb/V	0/2/2/0/0/0/1
Cholecystitis, *n*	2 (1%)
I/II/IIIa/IIIb/IVa/IVb/V	0/1/1/0/0/0/0
Bleeding, *n*	2 (1%)
I/II/IIIa/IIIb/IVa/IVb/V	0/0/2/0/0/0/0
Perforation, *n*	0 (0%)

**TABLE 4:** Risk factors associated with peritonitis after endoscopic ultrasound‐guided hepaticogastrostomy (EUS‐HGS).

	Univariate analysis		Multivariate analysis	
	Peritonitis, *n*	*p*‐value	Odds ratio (95% CI)	*p*‐value
Preprocedural cholangitis	7/116	0.57		
Total bilirubin level before HGS >10 mg/dL	1/33	0.47		
Diabetes	2/39	1.0		
Surgically altered anatomy	1/60	0.065	0.27 (0.031–2.4)	0.24
Malignant stricture	13/181	1.0		
Duodenal invasion	11/109	0.077	3.1 (0.61–16)	0.17
Peritoneal dissemination	2/43	0.74		
Ascites	2/33	1.0		
Punctured bile duct ≤3 mm	4/23	0.058	4.0 (1.1–15)	0.039
Distance to hepatic parenchyma <25 mm	6/59	0.23		
Bile aspiration <10 mL	2/56	0.35		
Tract dilation using dilation devices	11/168	0.63		
Electronic cautery catheter	6/87	1.0		
Plastic stent for HGS	3/60	0.56		
Without antegrade stenting	11/131	0.35		

Abbreviations: CI, confidence interval; HGS, hepaticogastrostomy.

## 4 Discussion

We investigated the factors associated with peritonitis following EUS‐HGS. The incidence of peritonitis was 7% in 188 patients who underwent EUS‐HGS. Multivariate analysis identified a punctured bile duct diameter ≤3 mm as an independent risk factor for peritonitis after EUS‐HGS.

The overall AE rate for EUS‐HGS was reported to be 13.7% in a 2023 meta‐analysis that included 7887 patients from 155 studies [20]. Furthermore, the incidence of peritonitis has been reported to range from 3.2% to 7.4% in previous prospective and retrospective studies [17, 21]. Our findings of a 15% overall AE rate and 7% peritonitis rate are comparable to those of previous reports.

Multivariate analysis of the factors associated with peritonitis identified a punctured bile duct diameter ≤3 mm as an independent risk factor. Although it may seem counterintuitive that a small bile duct diameter is a risk factor, similar findings have been reported. A single‐center retrospective study demonstrated that additional dilation was a factor associated with AEs of EUS‐HGS, and cases requiring additional dilation were shown to have bile duct diameters of ≤3 mm [22]. In the present study, the association between additional dilation and peritonitis was not statistically significant, likely because additional dilation was performed in the vast majority of cases as a standard institutional practice. However, theoretically, when the bile duct diameter is ≤3 mm, an additional step to insert a dilation device becomes necessary, leading to more device exchanges, which may increase the risk of bile leakage. Indeed, puncturing a bile duct ≤3 mm is anticipated to be difficult, as most fistula dilation devices have a diameter larger than the punctured duct, even if their tips are tapered. Therefore, once a guidewire is successfully inserted when puncturing a small bile duct, measures to reliably reduce the number of device exchanges, such as using an electrocautery dilator or a stent with a small‐caliber delivery sheath, may be warranted.

Meanwhile, although the *p*‐values were >0.05, factors such as bile aspiration of <10 mL before stent placement, omission of antegrade stenting, preprocedural hyperbilirubinemia, and duodenal invasion all had odds ratios >2, suggesting a clinically meaningful association. The involvement of the first two factors (aspiration and antegrade stenting) in AE development has been reported in previous retrospective studies [19, 23]. Bile aspiration presumably lowers intrabiliary pressure. As bile aspiration is performed routinely at our institution, cases with an aspiration volume of less than 10 mL represent patients with insufficient biliary dilation. In such cases, the intraductal pressure is expected to be relatively low, which may explain why bile aspiration did not significantly correlate with the incidence of peritonitis in this study. Similarly, antegrade stenting may reduce bile leakage from the HGS site by promoting biliary drainage into the duodenum before HGS stent placement. Hyperbilirubinemia has been reported to increase the risk of postoperative complications in pancreaticoduodenectomy [24, 25]; therefore, caution may also be warranted for pre‐HGS hyperbilirubinemia. Furthermore, regarding duodenal invasion, case reports have suggested that increased intragastric pressure before the fistula matures may contribute to peritonitis, highlighting another area for caution [26, 27]. Further large‐scale studies are needed to investigate these associations.

Although PSs have been implicated in AE development in earlier reports [28, 29], a recent single‐center retrospective study comparing PSs and MSs reported AE rates of 20% for MSs and 16% for PSs (*p* = 0.5) [30]. In the present study, PS use was not a significant factor. The availability of dedicated PSs in Japan may contribute to the improved AE rates for PSs.

This study had several limitations. First, this was a single‐center, retrospective study. The procedures were performed by or under the supervision of a single expert, resulting in a relatively uniform technique; however, generalizability may be limited, and the results might differ when the procedure is introduced at less experienced institutions. Second, there may be biases regarding the site of biliary stricture and the choice of the punctured bile duct. Furthermore, the majority of the original diseases were malignant. The possibility that these factors influenced the occurrence of peritonitis cannot be ruled out. Third, as the number of peritonitis cases was small (*n* = 13), the multivariate analysis remains exploratory. Fourth, there is no standardized consensus definition of peritonitis after EUS‐HGS, leading to variations across studies. Nevertheless, we defined peritonitis in a manner we believe is most clinically relevant, based on definitions used in previous research. Our 7% incidence of peritonitis is comparable to that in previous reports, but standardization of the definition will be crucial for future inter‐study comparisons. Fifth, although we aimed to capture new‐onset cases caused by EUS‐HGS, we cannot completely rule out the influence of pre‐existing ascites or the incidental occurrence of peritonitis from other causes, such as carcinomatous peritonitis, coinciding with the procedure. Finally, due to the retrospective and exploratory nature of this study, as well as the limited number of peritonitis events, we could not fully evaluate specific procedural complexity factors—such as device exchanges, tract manipulations, or dilation‐related factors—that might potentially contribute to bile leakage and peritonitis. Further prospective studies with more detailed procedural assessments are warranted to better elucidate the mechanisms underlying post‐EUS‐HGS peritonitis.

In conclusion, a punctured bile duct diameter of ≤3 mm was identified as a significant risk factor for the development of peritonitis in patients undergoing EUS‐HGS.

## Author Contributions

**Junya Sato** designed the research, analyzed the data, and wrote the manuscript. **Hirotoshi Ishiwatari** was primarily responsible for the final content. **Hiroki Sakamoto, Takuya Doi, Masahiro Yamamura, Kazunori Takada, Masahiro Yoshida, Sayo Ito, Noboru Kawata, Kenichiro Imai, Kinichi Hotta, and Hiroyuki Ono** contributed to the study concept, data acquisition, interpretation of the results, and revision of the manuscript. All the authors have read and approved the final manuscript.

## Funding

The authors have nothing to report.

## Ethics Statement

**Approval of the research protocol by an Institutional Reviewer Board**: Institutional ethical approval for this study was obtained from our facility's review board (J2022‐218).

## Consent

All patients provided informed consent for the procedure.

## Conflicts of Interest

The authors declare no conflicts of interest.

## Supporting information




**FIGURE S1**: Differences in punctured bile duct diameter with and without peritonitis after procedure.

**Supporting file 1**: deo270376‐supp‐0002‐Tables.docx
