# Supplementary material for: Risk Factors for Peritonitis Associated With Endoscopic Ultrasound‐guided Hepaticogastrostomy
Source: DEN Open. 2026 Aug 14;7(1):e70376. doi: 10.1002/deo2.70376 (PMC13474316; doi:10.1002/deo2.70376)
Supplement: Supplementary file 1 [file DEO2-7-e70376-s001.docx]

| **Supplementary Table 1. Relationship between punctured bile duct diameter and procedural factors** | | | |  |
| --- | --- | --- | --- | --- |
|  | Bile duct > 3mm  n = 165 | Bile duct ≤ 3mm  n = 23 | *P* value | |
| Punctured bile duct(B2), n | 74 (45%) | 9 (40%) | 0.66 | |
| Volume of aspirated bile, mL, median (IQR) | 25 (12-45) | 18 (0-27.5) | 0.087 | |
| Procedure time, min, median (IQR) | 30 (24-40) | 39 (25-46) | 0.22 | |
| Additional dilation, n | 149 (90%) | 19 (82%) | 0.28 | |
| Use of electronic cautery, n | 79 (48%) | 8 (35%) | 0.27 | |

| **Supplementary Table 2. Comparison of baseline characteristics by presence or absence of peritonitis after procedure** | | | |
| --- | --- | --- | --- |
|  | Peritonitis n = 13 | None-peritonitis  n = 175 | *P* value |
| Malignant biliary obstruction, n | 13 | 168 | 1.0 |
| Site of biliary obstruction (Hilar), n | 1 | 31 | 0.70 |
| Ascites, n | 2 | 31 | 1.0 |
| Peritoneal dissemination, n | 11 | 134 | 0.74 |
| Duodenal invasion, n | 11 | 98 | 0.077 |
| Surgical altered anatomy | 1 | 59 | 0.065 |
